# Author Index for Volume 95

**DOI:** 10.1038/sj.bjc.6603540

**Published:** 2006-12-12

Aapro, M 1467

Aasebø, U 966

Abbott, LH 1136

Abdel-Malek, ZA 496

Ablett, S 571

Achenbach, T 210

Adachi, G 862

Adachi, S 677

Adachi, Y 1653

Adam, PJ 298

Adams, DH 1202, 1545

Adema, GJ 896

Adenis, A 705

Aggarwal, R 21

Aglietta, M 13

Ahmad, T 581

Ahmed, AA 245

Ahn, JB 1637

Ahn, JS 1648

Ahn, M-J 1648

Ah-See, M 1683

Aihara, R 1642

Aikou, T 634

Aitchison, M 1076

Ajani, J 450

Akashi, A 817

Akslen, LA 366

Alabiso, I 13

Alabiso, O 13

Alain, T 1020

Albert, D 450

Alimonti, A 1304

Allen, N 406

Allhoff, EP 1167

Almonte, M 56

Altavista, P 49

Anand, S 485

Andersen, CL 1415

Anderson, H 822

Anderson, KC 961

Anderson, WF 123, 1302

Andersson, S 331

Andersson-Ellstrom, A 1459

Andrade, RP 1459

Andresen, O 966

Andrews, EJ 247

Andrews, L 1448

Andreyev, HJN 431

Androulakis, N 587

Angioli, R 699

Angst, E 307

Anlezark, G 1212

Anné, J 1212

Ansink, K 757

Anthoney, A 450

Apicella, C 1448

Araki, J 717

Aranda, S 667

Aravantinos, G 587

Arboine, D 1148

Arora, R 1250

Arriola, L 406

Arslan, A 1593

Arts, HJG 1125

Asahina, H 998

Asao, T 1642

Ashraf, N 1056

Aslanis, V 1161

Assersohn, L 1632

Atkin, GK 928

Attard, G 767

Atzpodien, J 463

Aubert, D 788

Auer, G 331

Aviel-Ronen, S 1148

Bach, SP 35

Bae, J 639

Baglioni, S 879

Bahre, R 210

Bailey, A 56

Bailey, D 841

Bain, M 649

Baldwin, C 431

Balladore, E 1101

Ballet, S 1326

Ballmer-Hofer, K 272

Bandobashi, K 1653

Bankier, A 1448

Bao, YP 96

Baptista, M 752

Baralle, D 233

Bardin, C 253

Bardin-Mikolajczak, A 123

Barget, N 1379

Barker, S 67

Barnetson, R 239

Baron, JA 363, 1609

Barr, E 1459

Barricarte, A 406

Barrow, D 172

Bartlett, JMS 627, 1234

Bartolazzi, A 204

Baryshnikova, E 1101

Basset-Seguin, N 548

Bataille, B 1062

Batistatou, A 674

Battelli, N 788

Bauer, J 710

Beaugrand, M 1379

Becker, N 378

Becket, E 1028

Beesley, AH 1537

Beijnen, JH 729, 794

Belldegrun, A 1455

Bellott, R 571

Benedetti Panici, P 699

Benesch, M 991

Bennett, DC 496

Bennink, EJ 896

Benusiglio, PR 1689

Benvenuti, S 879

Berardi, R 445

Berek, JS 1314

Beresford, MJ 1683

Bergelt, C 1579

Bergenheim, AT 766

Berghmans, T 139

Berglund, G 406

Bergman, B 966

Bernd, L 782

Berney, D 1186

Bernhardt, SL 1474

Bernhardtsen, T 416

Berrino, F 406

Berruti, A 13

Berthold, F 991

Bertorelle, R 1155

Besson, H 378

Beukenholdt, R 226

Bevilacqua, G 204

Bhattacharjya, S 21

Bhatti, LA 87, 423

Bhopal, R 424

Biade, S 1092

Bianchi, P 1101

Biasco, G 1525

Bibeau, F 1439

Bibi, R 1258

Biesma, B 470

Biesterfeld, S 210

Bilim, V 1244

Bing, C 1028

Bingham, S 406

Binlich, F 729

Bird, K 314

Birle, D 1148

Birtle, A 457

Biscuola, M 1155

Bitossi, R 13

Bjørge, T 366

Blackstock, AW 260

Bladström, A 986

Blair, A 123

Blair, CK 1274

Blanc, J-L 1062

Blatt, V 1155

Blay, J-Y 1326

Blázquez, C 197

Boddy, AV 1229

Bodmer, D 757

Bodo, J 1348

Boeing, H 406

Boerman, OC 896

Boffetta, P 378

Boggis, C 801

Boglino, C 49

Böhm, M 1167

Boldrini, R 49

Bolhuis, RJ 470

Bonichon, F 1529

Bonora, E 1529

Bontenbal, M 1334

Borthwick, A 1632

Bosanquet, N 6

Botti, G 1005

Boucher, E 705

Boukovinas, J 587

Bourbouloux, E 1161

Bourdon-Raverdy, N 944

Bourlard, T 788

Bourne, S 928

Boutreux, S 944

Boutron-Ruault, M-C 406

Bouvier, AM 944

Bouzourene, H 710

Bower, M 1145, 1709

Bowtell, D 829

Boyd, RS 298

Bozec, A 722

Bradstock, KF 181

Brandes, AA 1155

Bremnes, RM 966

Brennan, P 378

Brenton, JD 245

Brewster, DH 87, 423

Brice, A 548

Brinton, LA 123, 1302

Brizzi, MP 13

Brock, A 424

Broome, J 1424

Brown, C 398, 401, 660

Brown, J 801

Brown, JM 6

Brown, R 627

Brunelli, C 1121

Brunner, HG 1678

Brünner, N 1114

Brunstein, F 1663

Bryan, J 1459

Buanes, T 1474

Büchner, FL 406

Bueno-de-Mesquita, HB 406

Bulten, J 1126

Bundred, NJ 1410

Burger, HU 1467

Burke, PJ 1212

Byron, SA 1220

Cadirni, A 153

Cai, L 1455

Calcagno, M 699

Caldas, C 245

Cameron, D 1626

Camidge, DR 649

Campagna, S 153

Campagnutta, E 699

Campbell, H 239

Campbell, M 435

Campbell, R 1234

Camplejohn, RS 520

Campone, M 1161

Candinas, D 307

Cao, W 1455

Cao, Y 1611

Cappelli, CA 49

Caraceni, A 1121

Carlsen, K 653

Carneiro, F 406

Carpenter, B 921

Carpi, A 204

Carstensen, H 416

Carter, N 520

Cascinu, S 445

Case, LD 260

Cassidy, A 1288

Cassoni, A 1308

Castedo, S 752

Catalano, V 445

Cavallo, G 1155

Cavassini, M 1598

Cebon, J 853

Cebrian, A 525

Ceccarelli, C 1525

Cellai, I 879

Ceni, E 879

Cerny, T 1342

Cervera, N 1439

Chan, A 788

Chan, JK 1314

Chandanos, E 118

Chang, S-F 1384

Chang, S-H 639

Chang, Y 485

Chantereau, T 1062

Chao, TY 159

Chao, Y 159

Chaoui, D 253

Charalabopoulos, A 674

Charalabopoulos, K 674

Chast, F 253

Chastagner, P 1326

Chatrath, P 314

Chatterjee, N 123

Chauhan, D 961

Chen, C-A 1384

Chen, C-Y 282

Chen, LT 159

Cheng, AL 159

Cheng, J-R 1586

Cheng, W-F 1384

Cheng, Y 1379

Cheong, SC 496

Cheung, ALM 475

Cheung, ANY 1087

Cheung, H-W 475

Cheung, MK 1314

Chevret, S 253

Chiari, S 699

Chick, J 450

Chidgey, M 1367

Chilcott, J 1195

Chinnaiyan, AM 425

Chirlaque, MD 406

Cho, BC 1637

Chong, H 496

Chow, J 496

Christensen, IJ 1114

Christinat, A 1342

Christoph, F 1701

Chu, T-Y 1384

Chuang, M-H 1384

Chuchana, P 1439

Chung, HC 1637

Ciruelos, E 1161

Clark, L 593

Clarke, A 435

Clarke, CL 515

Clarke, RB 1410

Clavel-Chapelon, F 406

Clifford, GM 96, 1593, 1598

Cocco, PL 378

Coebergh, JWW 393

Cognetti, F 763, 1304

Cohen, PS 829

Coiffier, B 1467

Colarossi, C 49

Cole, BF 1603

Colebatch, A 1239

Coleman, MP 1296

Coleman, N 314

Colin, C 1161

Collard, JG 1081

Collecchi, P 204

Collins, L 282

Collins, R 457

Colombo, A 266

Colpaert, CG 1362

Comella, G 1005

Comella, P 1005

Conroy, D 1689

Copeland, J 6

Copeman, M 829

Cordon-Cardo, C 1455

Cortes-Funes, H 1161

Costa, RLR 1459

Costes-Martineau, V 355

Coucke, P 710

Cowan, J 27

Cozzi, D 49

Craigs, C 979

Cramer, A 1410

Cremer, FW 782

Crickx, B 548

Crijns, APG 627

Crinò, L 1155

Croft, S 835

Crown, J 1626

Cui, Z-G 1371

Cummings, AJ 1537

Cummings, J 42

Curigliano, G 289

Curtis-Snell, L 616

Cuzick, J 1186

Czene, K 1291

Dahan, L 705

Dai, DL 80

Dai, M 96, 1593

Dai, Q 1586

D’Aiuto, G 1005

Dal Maso, L 1598

Dalbagni, G 1455

Daley, F 1683

Daley, FM 928

Dallimore, N 226

D’Alo’, F 1108

Dalton, SO 653, 934

Daly, MB 1448

Damdinsuren, B 889

Dancey, J 1148

Danesi, R 289

Danzon, A 944

Das, BC 1250

Davidsen, ML 1114

Davidson, BR 21

Davies, E 593

Davies, RJ 314

de Bock, GH 627, 1125

de Bono, JS 767

De Cobelli, B 289

De Conno, F 1121

De Fraud, F 289

de Graeff, P 627

de Hullu, JA 1126

de Jong, S 627

de Klerk, NH 1537

De Lisi, V 1265

De Pouvourville, G 944

De Rosa, V 1005

De Sanjosé, S 378

de Vries, EGE 1334

Deans, DAC 1568

Dejardin, O 944

Del Giudice, G 406

Del Tacca, M 289

Delafosse, P 944

Delattre, O 1326

Delgado, FM 1161

Dell’Anna, T 699

den Brok, MHMGM 896

Denis, S 1062

Desai, M 56

Descamps, V 548

Descos, L 260

Destro, A 1101

Dew, G 1689

Dewhirst, MW 1, 1013

Dewilde, S 683

Di Bonito, M 1005

Di Fiore, F 705

Didelot-Rousseau, M-N 355

Dietel, M 339

Dietrich, D 1342

Dirix, LY 1362, 1611

DiRuscio, A 1108

Dive, C 42, 1410

Dixon, A 801

Dixon, M 801

Dogliotti, L 13

Doll, D 1419

Dominici, C 49

Dominkus, M 691

Donfrancesco, A 49

Donovan, JL 1122

Dormans, JP 1608

Dosaka–Akita, H 998

Douglass, C 1603

Downing, A 91

Dowsett, M 661

Doz, F 571

Drolet, DW 450

du Boulay, C 841

du Plessis, DG 1424

Duarte, MJ 197

Dubois, L 1212

Duffy, GJ 1174

Duffy, SW 398, 401, 660, 1288

Dundas, SR 921

Dunlop, M 239

Dunning, A 1689

Dunning, AM 525

Dunst, J 1467

Dupin, N 548

Duraj, J 1348

Duran, I 1148

Düring, M 653

Durkin, J 42

Dûrre, P 1212

Dusart, M 139

Dyrskjøt, L 1415

Dzięgiel, P 339

Eastham, J 1186

Easton, D 233, 801

Easton, DF 525, 1689

Eatock, M 450

Ecke, M 1167

Edwards, S 1545

Eeles, R 801

Egerer, G 782

Eggermont, AMM 1663

Eggington, S 1195

Eguchi, S 561

Eijkemans, MJC 1180

Eisen, T 581

Elfgren, K 1459

Ellis, IO 735

Elsworth, A 571

Emuss, V 1128

Engeland, A 366

Engellau, J 986

Engels, EA 642

Engers, R 1081

English, WR 245

Eplov, LF 146, 1610

Erb, P 1598

Eriksen, JA 1474

Ermani, M 1155

Esser, MT 1459

Evangelisti, C 1529

Evangelou, A 674

Evans, DG 801

Fabian, I 1038

Fabiani, E 1108

Falcini, F 1265

Falk, S 450

Fan, S-T 1050

Farrington, SM 239

Faulkner, R 667

Favalli, G 266

Fearn, P 1186

Fearon, KCH 1568

Federico, M 1265

Felici, A 763

Felix, CA 1274

Fenwick, E 457

Fernebro, J 986

Ferrand, S 1062

Ferrari, P 406

Ferretti, G 763, 1304

Ferretti, S 1265

Ferru, A 1670

Ferry, D 450

Ferry, DR 835

Fey, MF 1342

Fiander, AN 226

Field, JK 1288

Fietkau, R 951

Figdor, CG 896

Figlin, RA 1455

Fildes, D 1424

Firer, MA 189

Firth, MJ 1537

Fischbacher, CM 424

Fischel, J-L 722

Fisher, G 1186

Flaherty, KT 581

Fletcher, GC 298

Floriani, I 699

Font, R 378

Ford, J 1537

Foretova, L 378

Forman, D 91, 979

Formento, P 722

Fornara, P 463

Forsyth, PA 1020

Forti, L 13

Fossati, R 266, 699

Foster, CS 1186

Foster, E 1626

Foulkes, WD 243

Fountzilas, G 587

Fox, J 1490

Fox, KF 1454

Fox, SB 1611

Fragoso, M 752

Franceschi, E 1155

Franceschi, S 96, 1593, 1598

Franchi, G 1101

Francini, E 153

Francini, G 153

Franks, PJ 6

Frappaz, D 571

Frasci, G 1005

Fraser, L 1124

Frayling, I 233

Freitas, JR 1537

Frerichs, K 210

Friederichs, J 1419

Frielink, C 896

Friesen, M 406

Friis, S 363, 934, 1609

Fromont, G 1670

Fujino, H 75

Fujita, Y 998

Fujiwara, M 1504

Fujiwara, Y 1390

Fukuoka, M 677

Fukushima, M 607

Fumoleau, P 1161

Fung, MKL 475

Fux, C 1598

Gabbert, HE 1081

Galais, MP 705

Galizia, E 445

Galle, PR 210

Galli, A 879

Galve-Roperh, I 197

Ganganagari, JR 42

Gao, Y-T 1586

García-Closas, M 123, 1302

Garnero, P 506

Garrigue, JS 729

Gasmi, J 788

Gatter, KC 298

Gaudernack, G 1474

Gee, JMW 172

Geissler, M 848

Gemba, K 1142

Gennari, L 1101

George, LM 112

George, S 841

George, WD 1056

Georgoulias, V 587, 788

Gerard, B 548

Gertenbach, U 463

Giacoletti, K 1459

Gibbens, I 581

Gibelin, H 1670

Gil, MG 788

Gilbert, FJ 801

Gilham, C 56

Gilleryd, M 966

Gillham, CM 1174

Gilliland, G 554

Gilmour, H 1568

Gilthorpe, MS 979

Gioia, V 1155

Giordani, P 445

Giovannetti, E 289

Giovannucci, E 1277

Gjerris, F 416

Gjertsen, MK 1474

Glen, H 450

Gloor, B 307

Gloudemans, S 757

Glynne-Jones, R 928

Gnekow, AK 991

Gockel, I 210

Goedert, JJ 642

Goekkurt, E 1306

Goldacre, M 940

Goldbohm, RA 374

Goldschmidt, H 782

Gollin, SM 1432

Gologan, A 243

Gonnelli, S 153

Gonzalez, CA 406

González-Feria, L 197

Goossens, M 1678

Gore, M 581, 829

Gorin, I 548

Gorzegno, G 13

Goss, G 1148

Götte, M 347

Gottlieb, DJ 181

Goudie, D 974

Gould, A 1626

Granath, F 1291

Grandchamp, B 548

Grandis, JR 164

Grant, A 435

Grassi, R 699

Gray, J 435

Gray-Schopfer, VC 496

Graziano, F 445

Greggi, S 699

Griebsch, I 801

Griffioen, G 744

Griffiths, C 424

Grønbæk, M 1579

Grosche, B 1280

Grosclaude, P 944

Gugenheim, J 722

Guidi, F 1108

Guilhot, J 1062, 1670

Guillem-Porta, V 788

Guillou, L 1342

Guillou, PJ 6

Gumiero, D 1108

Guo, J 282

Gustafson, P 986

Guzmán, M 197

Gwack, J 639

Györffy, B 339

Hackett, S 581

Haga, N 1642

Hagiwara, K 1483

Haitel, A 691

Haites, N 435

Hall, J 627

Hall, P 1291

Haller, A 139

Halperin, D 1038

Hamdy, F 1122

Hamer, O 1608

Hamidou, H 705

Hamilton, W 1321

Hammarström, M-L 218

Hammarström, S 218

Hannemann, J 1334

Hansen, PE 146, 1610

Haq, A 1367

Hara, N 1244

Harada, M 998

Harder, J 848

Hardy, R 1367

Harris, A 13

Harris, AL 298, 1611, 1683

Hart, K 226

Hartge, P 107

Hasan, T 485

Hasse, B 1598

Hatano, H 1396

Hatch, EE 107

Hauke, A 226

Hauser, P 554

Hauser, S 463

Haward, RA 979

Hawkins, NJ 1239

Hawnaur, J 801

Haylock, B 1424

He, G 1514

Hebbar, S 869

Hebeda, KM 1678

Hedley, D 1148

Heinzer, H 463

Hejna, M 691

Helgason, HH 794

Hellot, MF 705

Hellström, A-C 331

Henß, H 848

Henriksson, R 766

Herbst, AL 107

Herndon II, JE 1013

Herrmann, R 1342

Heselmeyer-Haddad, K 331

Hessol, NA 642

Heynemann, H 463

Hibbitts, S 226

Hideshima, T 961

Higashimoto, K 541

Hill, ADK 1404

Hilmy, M 1234

Hiraki, S 1142

Hirte, H 1148

Hitchins, M 1239

Hjerpe, A 331

Ho, C-M 1384

Ho, J 1148

Ho, V 80

Hodgson, S 520

Hofland, LJ 1497

Hofman, P 722

Hohaus, S 1108

Hollema, H 627

Holloway, B 172

Hollywood, D 1174

Holzmann, B 1419

Honegger, HP 1342

Hong, Y-C 639

Hong, YS 1648

Hoogerbrugge, N 757, 1126, 1678

Hoover, RN 107, 1603

Hopper, JL 1448

Hörner, V 210

Horwitz, KB 1220

Houghton, PJ 955

Houlston, RS 1047

House, C 829

Høye, J 1459

Hsieh, C-Y 1384

Hsieh, RK 159

Huang, C 607

Huang, RD 96

Huang, S 955

Huang, Z 1514

Hubscher, SG 1202

Huitema, ADR 729, 794

Huland, E 463

Hulsbergen-van de Kaa, CA 374

Humphreys, A 1626

Hunakova, L 1348

Hupperets, P 1334

Hurt, C 1490

Husain, A 1314

Husband, D 1424

Huygelen, V 1362

Hwang, IG 1648

Hyer, M 107

Hyodo, A 862

Iacopetta, BJ 1708

Ichinose, Y 717

Iervasi, G 204

Ieta, K 532

Iguchi, C 811

Ikeda, M 889

Ikehara, S 1504

Ikehara, Y 1504

Ikezoe, T 1653

Ino, K 1555

Inoue, O 862

Inoue, R 561

Inoue, Y 717

Iodice, G 1005

Iraha, S 862

Ishida, T 998

Ishigami, S 634

Isobe, H 998

Israelsson, A 218

Itami, A 322

Ito, H 561

Ito, K 717

Ito, T 322

Itoi, T 1244

Ittenson, A 1167

Iuchi, K 817

Iversen, O-E 1459

Iveson, T 131

Iyer, NG 245

Jack, RH 593

Jackson, DG 1611

Jacob, JH 705

Jacobs, IJ 1124

Jakubikova, J 1348

James, M 581

Jamieson, D 1229

Janssen, KP 1419

Jayson, G 42

Jeekel, J 1497

Jenab, M 406

Jenkins, JR 1028

Jenkinson, MD 1424

Jensen, T 416

Jerónimo, C 752

Jeung, H -C 1637

Jia, C 118

Joh, K 541

Johansen, C 146, 653, 934, 1579, 1610

Johansen, SK 1114

Johansson, A 406

Johansson, I 406

Johansson, M 766

Johnson, PJ 1379

Johnson, SW 1092

Johnston, RN 1020

Jones, AA 515

Jones, HE 172

Jordan, C 1689

Jordan, H 841

Joshipura, K 1603

Joyce, KA 1424

Juillet, F 506

Jun, JK 639

Junginger, T 210

Kaaks, R 406

Kachnic, LA 260

Kaganoi, J 322

Kailash, U 1250

Kajiyama, H 1555

Kakinohana, Y 862

Kalaylioglu, Z 123

Kalebic, T 506

Kalfakakou, V 674

Kalk, E 1202

Kaltenthaler, E 27

Kameda, Y 1070

Kamei, H 1142

Kamiyama, Y 1642

Kan, C-Y 1708

Kan, N 811

Kanazawa, M 1483

Kanazawa, S 1142

Kanazawa, T 1562

Kaneko, Y 541

Kang, D 639

Kang, HH 914

Kanold, J 1326

Kao, WY 159

Kapp, DS 1314

Karapetis, CS 1309

Karayan-Tapon, L 1062, 1670

Kasahara, K 1390

Kasparkova, J 1348

Kato, BS 520

Kattan, MW 1186

Kaufman, R 107

Kawaguchi, T 601

Kawakatsu, H 1410

Kaye, S 581

Kazama, S 1562

Kazama, Y 1562

kConFab Investigators 914

Keefe, DM 1309

Kees, UR 1537

Kellokumpu-Lehtinen, P 788

Kelly, G 1404

Kempkensteffen, C 1701

Kenzaki, K 75

Keogan, M 1174

Kerob, D 548

Kersting, C 347

Kessar, P 801

Kets, CM 1678

Key, T 406

Khan, K 1367

Khanna, M 243

Khaw, K-T 406

Khokhar, AR 1514

Kidokoro, K 1555

Kiemeney, LALM 374

Kiesel, L 347

Kikkawa, F 1555

Kikuchi, N 371

Killeen, SD 247

Kim, C-S 639

Kim, M 1020

Kim, Y 639

Kimura, H 1390

Kimura, S 1354

Kingsmore, D 1056

Kinlen, L 102

Kinoshita, I 998

Kirchner, H 463

Kirk, J 914

Kita, Y 634

Kitagawa, M 1396

Kitajima, S 1396

Kitchener, HC 56

Kiura, K 1142

Klatte, T 1167

Klautke, G 951

Knox, F 1410

Knox, RJ 1229

Ko, K-P 639

Kobayashi, K 1483

Kobayashi, M 1653

Kobayashi, R 1514

Koch, U 1579

Kocha, W 1148

Koczwara, B 1309

Kodama, H 811

Kodama, K 817

Kodera, Y 1504

Koeffler, HP 1653

Kofler, A 1598

Koh, KA 1582

Koike, M 1504

Koizumi, F 601

Kojima, T 998

Kokai, G 49

Köllermann, J 1701

Konate, I 355

Kondo, K 75

Koomen, J 1514

Kooshki, M 869

Kopitzki, K 1424

Kordek, R 123

Kornafel, J 339

Kortmann, R-D 991

Kortmansky, J 1148

Kossakowska, AE 1020

Kotasek, D 1309

Kotsakis, A 587

Kotsalos, A 674

Kötz, BS 835

Koyama, N 1483

Kraimps, JL 1670

Krause, H 1701

Kreisheimer, M 1280

Kreuzer, M 1280

Kridel, S 869

Kristel, P 1334

Kristiansen, G 339

Krubasik, D 245

Kruijtzer, CMF 794

Kuang, J 1514

Kubben, FJGM 744

Kudo, Y 1396

Kudoh, S 677

Kuipers, EJ 1180

Kummer, O 848

Kunitoh, H 1390

Kuriyama, S 371

Kurmasheva, RT 955

Kuroda, J 601

Kuwano, H 532, 1642

Kvasnicka, P 1348

La Vecchia, C 390

LaCasse, E 42

Lademann, U 1114

Lafitte, J-J 139

Lage, H 339

Lagergren, J 118

Laghi, L 1101

Lakhani, SR 515, 801

Lakshmy, R 1250

Lallemand, F 729

Lalor, PF 1545

Lamb, GWA 1076

Lambert, G 729

Lambin, P 1212

Lamers, CBHW 744

Lamy, P 1415

Landoni, F 699

Landuyt, W 1212

Lane, JA 1122

Lange, CA 1220

Lansdown, M 1301

Lapierre, F 1062

Laptev, R 189

Larocca, LM 1108

Larsen, C-J 1062, 1670

Larsen, JK 1114

Larsson, C 331

Larsson, SC 1277

Lasorsa, L 1439

Lassalle, S 722

Last, K 1632

Latz, JE 677

Launoy, G 944

Laurence, V 1326

Lavra, L 204

Lawrie, LC 921

Lawson, JS 1708

Le, L 1148

Le Morvan, V 906

Leach, BI 298

Leach, MO 801

Leary, A 661, 1632

Leary, J 914

Lebbe, C 548

Ledergerber, B 1598

Lee, HR 1648

Lee, I-M 1582

Lee, J 1648

Lee, PWK 1020

Lee, SC 1648

Lee, TK 1050

Legrand, O 253

Lehtinen, M 1459

Leiber, C 463

Leigh, Y 940

Leiserowitz, GS 1314

Lejars, O 1326

Leonard, RCF 1626

Leone, G 1108

Lepage, C 1296

Lesueur, F 1689

Levi, F 390

Levine, EA 260

Levitov, A 1038

Lévy, V 253

Lewis, I 571

Leygue, E 616

Leyvraz, S 710, 1342

Li, CP 159

Li, C-Y 1696

Li, F 1696

Li, G 80

Li, LK 1593

Li, N 96, 1593

Li, Y-C 181

Liboutet, M 548

Lieske, B 62

Light, K 457

Ligtenberg, MJL 757, 1126, 1678

Liloglou, T 1288

Lim, HY 1648

Limentani, SA 260

Lindblad, M 118

Lindmark, G 218

Ling, M-T 475

Linklater, KM 593

Linseisen, J 406

Lionello, I 181

Lissoni, A 266, 699

Lissowska, J 123

Little, J 869

Liu, D 607

Liu, VWS 1087

LiVolsi, VA 1092

Lloyd, A 683

Lo, C-M 1050

Loader, JA 298

Lohrmann, C 848

Longavenne, E 1326

Longy, M 906

Lopes, C 752

Lorigan, P 822

Losty, PD 49

Lothaire, P 139

Louwman, WJ 393

Love, S 1490

Lowis, S 571

Lu, F 80

Lu, L-S 298

Lu, ML 1455

Luben, RN 1689

Lucey, JA 1174

Luciani, P 879

Lukaszek, S 123

Lund, E 406

Lupinacci, L 1459

Lynch, J 653

Lynch, K 829

Maarouf, N 944

Maciejczyk, A 339

MacIntyre, A 1056

Mackay, J 1124

Maekawa, T 1354

Maggi, R 266

Maggioni, A 699

Magnino, A 13

Mai, Z 485

Makris, A 1683

Malesci, A 1101

Malik, V 1174

Malik, Z 189

Malinovszky, KM 1626

Malm, C 1459

Man, K 1050

Manci, N 699

Mang, OW-K 1269

Mangioni, C 699

Mangone, L 1265

Mannarino, O 49

Mano, MP 1265

Marais, R 496, 581

Marangolo, M 181

Maraqa, L 1301

Marchetti, C 204

Marcus, SG 794

Mareau, E 506

Marec-Berard, P 1326

Margison, GP 822

Margison, JM 822

Mari, D 445

Marie, JP 253

Marinucci, M 1092

Marmorale, C 445

Marples, M 27

Martin, A 1379

Martin, B 139

Martin, F 822

Martin, M 788, 1161

Martini, C 1121

Martini, M 1108, 1419

Martinka, M 80

Marty, C 272

Maruyama, R 1244

Masaki, Z 541

Mascaux, C 139

Mason, S 298

Massuger, LFAG 1126

Masters, MN 1603

Masuya, D 607

Materna, V 339

Matkowski, R 339

Matsui, K 677

Matsukuma, S 1070

Matsumoto, H 561

Matsumoto, M 634

Matsumura, Y 601

Matsuo, K 1142

Matsuura, N 889

Matsuyama, H 561

Matter, M 710

Mattsson, B 347

Mayaud, P 355

Maynadié, M 378

Maytin, EV 485

Mazepa-Sikora, D 123

McCulloch, P 940

McDermott, EW 1404

McDowell, HP 49

McKay, M 921

McLaughlin, JK 934

McLeish, L 974

McMillan, DC 1076, 1234

McNeel, TS 642

McNicol, A-M 1234

McRea, J 226

Meagher, A 1239

Meert, A-P 139

Meijer, CJLM 96, 1593

Meijer, MJW 744

Melin, SA 260

Melpignano, M 266

Mendiola, C 1161

Mengesha, A 1212

Menon, U 1124

Meo, M 1474

Mesquita, B 752

Metcalfe, C 1122

Metzner, B 463

Mey, V 289

Michel, P 705

Michon, J 1326

Michor, F 1136

Miedzybrodzka, Z 435

Mik, G 1608

Milan, S 1632

Milano, G 722

Milin, S 1062

Miller, K 1701

Millward, MJ 829

Milne, D 667

Minton, NP 1212

Mirabel, X 705

Miraglia, S 13

Miranda, E 1101

Mishra, G 260

Miwa, S 1371

Miyagi, Y 1070

Miyauchi, M 1396

Miyazawa, H 1483

Miyoshi, T 75

Mochiki, E 1642

Mochizuki, Y 1504

Modugno, F 1432

Moehler, M 210

Moehler, T 782

Moeller, BJ 1

Moffa, IF 1265

Moisan, F 906

Moldovan, S 1309

Molife, R 767

Molinié, F 944

Møller, H 593, 1186

Møller, M 1474

Mollison, J 435

Monden, M 889

Montagnani, A 153

Moreira-Dias, L 752

Morenghi, E 1101

Mori, M 532

Mori, Y 322

Moritaka, T 1142

Mornex, F 260

Morris, E 979

Morris, JK 112

Morris, LS 314

Morrison, LE 331

Mortensen, PB 653, 934

Morton, D 1367

Moss, PA 1202

Moss, SM 801

Moss, T 496

Mourits, MJE 1125

Mueller, BU 554

Mueller, M 1081

Mukai, T 541

Muldoon, C 1174

Mullee, M 841

Murai, Y 1371

Muratori, M 879

Murayama, S 862

Murayama, Y 1483

Murphy, G 245

Murphy, LC 616

Murray, GI 921

Murray, JC 735

Myers, E 1404

Myles, JP 1288

Mymryk, JS 555

Naccarato, AG 204

Nafees, B 683

Nagai, Y 1483

Nagasaka, T 1555

Nagata, M 1483

Nagawa, H 1562

Nagel, G 406

Nagot, N 355

Naito, K 561

Naito, S 601

Nakabayashi, T 1642

Nakadate, H 541

Nakagawa, K 677, 817

Nakagawachi, T 541

Nakamura, J 1642

Nakanishi, H 1504

Nakano, J 607

Nakao, A 1504

Nakashima, T 607

Nakaya, N 146, 371, 1610

Naldoni, C 1265

Nannizzi, S 289

Narewska, J 683

Naska, A 406

Näslund, L 218

Natali, PG 49

Nathanson, KL 581

Natsugoe, S 634

Nawa, A 1555

Neal, D 1122

Negishi, T 601

Neglia, JP 1274

Negoro, S 677

Nerurkar, A 801

Neuberg, D 554

Neviani, P 775

Newton-Bishop, JA 91

Ng, KT 1050

Ngan, CY 889

Ngan, HYS 1087

Nguyen, C 1439

Nicholson, RI 172

Nicklee, T 1148

Nicolini, A 204

Nicotra, MR 49

Nierkens, S 896

Nieters, A 378

Nilbert, M 986

Nio, Y 811

Nishimura, M 998

Nishino, Y 371

Nishio, K 1390

Nishioka, C 1653

Nishiyama, T 1244

Nisnevitch, M 189

N’Kontchou, G 1379

Noakes, KL 1202

Nogawa, M 1354

Nol, A 729

Noller, K 107

Nomoto, K 1371

Nomura, S 1555

Nooij, MA 1334

Norat, T 406

Nørgaard, M 934

Norman, G 457

Northover, JMA 928

Nugoli, M 1439

Nuijen, B 729

Numans, ME 406

Öberg, Å 218

Oberlin, O 1326

O’Brien, MER 1632

O’Byrne, K 1174

Odermatt, B 272

O’Dwyer, PJ 581, 1092

O’Dwyer, ST 35, 1258

Oei, ALM 1126

Oestergaard, MZ 525

Offenberg, H 1114

Ogawa, I 1396

Ogawa, K 862

Ogunbiyi, O 1367

Ogura, S 998

Ohgane, N 1070

O’Higgins, NJ 1404

Ohlsson, L 218

Ohmi, C 561

Ohmori, K 371

Ohno, T 1642

Ohsawa, H 1642

Oien, KA 627

Oikonomou, E 406

Ojima, H 1642

Okamoto, Y 998

Okumura, H 634

Okumura, T 322

Oliver, D 953, 1129

Oliver, T 1186

Olsen, A 406

Olsson, H 986

Olsson, S-E 1459

Olver, IN 829, 1309

Oosterwijk, E 374

Oosterwijk, JC 757

Opitz, O 848

Ørntoft, T 1415

Orr, B 27

Orsetti, B 1439

Ortel, BJ 485

Osann, K 1314

Oshita, F 1070

Österborg, A 1467

Ottanelli, B 879

Otto, F 848

Ouedraogo, A 355

Overvad, K 406

Owaki, T 634

Oza, A 1148

Ozakinci, G 974

Paavonen, J 1459

Pabst, T 554

Paci, E 1265

Padhani, AR 801, 1683

Paesmans, M 139

Paffenbarger Jr, RS 1582

Paillot, B 705

Painter, R 385

Paisley, S 1195

Palli, D 406

Palmer, JR 107

Palmer, M-L 1537

Palmer, S 457

Paluchowski, P 339

Pandor, A 1195

Panico, S 406

Pantaleo, MA 1525

Pantuck, A 1455

Papagrigoriadis, S 950

Papakotoulas, P 587

Park, B-B 1648

Park, K 1648

Park, S 1648

Park, SK 639

Parker, C 767

Parnis, FX 1309

Partensky, C 260

Pashayan, N 398, 401, 660, 1296

Patani, NR 515

Paterini, P 1525

Paterson-Brown, S 1568

Patkar, V 1490

Patterson, A 431

Patterson, WK 1309

Paul, J 627

Paul, MA 470

Pawlicki, M 788

Payne, AP 1056

Peacock, S 1448

Pece, S 289

Pedersen, L 363, 1609

Peeters, PH 406

Pein, F 571

Pellegrino, A 699

Pennington, O 1212

Peplonska, B 123

Pepper, MS 1611

Pera, G 406

Perera, KU 1537

Peri, A 879

Perla, FM 49

Perrotti, D 775

Peschos, D 674

Pestalozzi, B 1342

Peter, M 1326

Peters, AM 1274

Peters, TJ 1321

Peto, J 56

Petrioli, R 153

Petruzelka, L 788

Petta, CA 1459

Pettersson, F 766

Pezzella, F 298

Pharoah, PD 1689

Pharoah, PDP 525

Phernambucq, ECJ 470

Piazzi, G 1525

Pierron, G 1326

Pietsch, T 991

Pinheiro, M 752

Pinkerton, R 571

Pinto, C 752

Pitkethly, M 974

Pittman, KB 1309

Plebani, M 406

Pointon, LJ 801

Polesel, J 1598

Polyzos, A 587

Pond, GR 1148

Ponder, B 233

Ponder, BAJ 525

Ponder, BAJ 1689

Ponder, M 233

Ponti, A 1265

Poon, RT 1050

Pope, M 1028

Portela, M 548

Porteous, M 239

Postmus, PE 470

Potamianou, A 587

Potten, CS 35

Potterton, J 801

Poulin-Costello, M 581

Poulsen, A 363, 1609

Poulsen, AH 934

Powell, NG 226

Powles, J 398, 401, 660

Powles, T 1145, 1709

Pranesh, N 1258

Prescott, E 1579

Price, TJ 1309

Priel, E 1038

Primrose, J 841

Probst-Hensch, NM 1598

Prochazka, M 1291

Prod’homme, S 705

Prosser, J 1424

Puddefoot, JR 67

Pudełko, M 339

Puliti, D 1265

Purushotham, A 1490

Qiao, YL 96, 1593

Quirke, P 6

Quirós, JR 406

Raaschou-Nielsen, O 416

Rabbani, ZN 1013

Rabkin, CS 642

Rachet, B 1296

Radke, I 347

Raffoux, E 253

Ragin, CCR 1432

Railkar, R 1459

Ramsey, S 1076

Randimbison, L 390

Ranson, M 42, 822

Rao, JY 1455

Raouf, AA 1174

Raoul, JL 705

Raschke, R 463

Ratain, MJ 581

Ravichandran, D 62

Ravina, J 197

Reaman, GH 1274

Rebmann, U 463

Redmond, HP 247

Reid, A 767

Reis, M 974

Reitz, M 463

Remontet, L 944

Renehan, AG 1258

Rens, J 1663

Reuter, V 1186, 1455

Reuveni, D 1038

Reynolds, GM 1202

Reynolds, JV 1174

Rha, SY 1637

Riboli, E 406

Richer, JK 1220

Rickenbach, M 1598

Ricker, W 107

Rieck, GC 226

Riecken, B 848

Ried, T 331

Riemsma, R 457

Rigg, A 1632

Riis-Johannessen, G 1459

Rimkus, C 1419

Ringland, C 914

Rio, B 253

Robbins, M 869

Robert, J 571, 906

Roberts, D 1092

Robison, LL 1274

Rodenhuis, S 1334

Rodriguez, C 1439

Roh, JK 1637

Roh, WJ 1637

Röhl, F-W 1167

Roigas, J 463

Romeo, G 1529

Rømer, MU 1114

Roncalli, M 1101

Rosa, MC 197

Rosenberg, R 1419

Rosenthal, AN 1124

Roskelley, C 245

Ross, JA 1274, 1568

Ross, L 653

Rossi, RS 699

Rossie, KM 1432

Rougé, C 1439

Roumen, RMH 393

Rousselot, P 253

Ruan, Z-X 1586

Rubio, CA 118

Rubulotta, MR 1005

Rudd, MF 1047

Ruers, TJM 896

Russell, S 1028

Russo, V 181

Rutkowski, S 991

Rydholm, A 986

Rymkiewicz, G 123

Saah, AJ 1459

Sabate, J 406

Sabbadini, RA 1131

Sabo, D 782

Sacerdote, C 406

Sage, EH 1092

Saiag, P 548

Saini, A 13

Saito, A 862

Saito, H 1070

Saito-Nakaya, K 146, 1610

Sakai, Y 322

Sakano, S 561

Sakiyama, S 75

Sakuma, Y 1070

Salm, S 463

Sammit, A 1145

Samonis, G 587

Sánchez, C 197

Sánchez, M-J 406

Sandle, J 515

Sanitt, A 1709

Santos, L 752

Sargent, A 56

Sarker, D 767

Sato, K 1354

Satoh, Y 541

Sauleau, EA 944

Saunders, M 131, 1195

Saunders, MP 1258

Savage, PM 1145, 1709

Savagner, F 1670

Save, V 406

Scambia, G 699

Scardino, P 1186

Scardocci, A 1108

Scarpino, S 49

Scartozzi, M 445

Schairer, C 642

Schalken, JA 374

Schapiro, IR 146, 1610

Schellens, JHM 729, 794

Schick, J 1092

Schimanski, CC 210

Schleiermacher, G 1326

Schmid, P 1145, 1709

Schmidinger, M 691

Schmiegelow, K 416

Schnelzer, M 1280

Schofield, P 667

Schornagel, JH 794

Schostak, M 1701

Schot, ME 794

Schouten, LJ 374

Schrader, M 1701

Schramel, FM 470

Schuchter, LM 581

Schuler, M 210

Schulz, M 406

Schwartz, B 581

Schwendener, RA 272

Schwindl, B 463

Sciacchitano, S 204

Scott, IS 314

Sculier, J-P 139

Sculli, CM 13

Seagroatt, V 940

Seckl, MJ 1145, 1709

Sedlak, J 1348

Segawa, H 1354

Segawa, Y 1142

Segondy, M 355

Sekimoto, M 889

Semba, H 717

Sentenac, S 253

Serio, M 879

Sessa, C 1342

Sesso, HD 1582

Seto, T 717

Setoyama, T 634

Seymour, M 1195

Seymour, MT 27

Shah, M 525

Shalit, I 1038

Sharp, D 1321

Sherman, ME 123, 1302

Shi, JF 1593

Shibata, K 1555

Shibayama, T 1142

Shiels, PG 1056

Shimada, Y 322

Shimazu, T 371

Shin, A 639

Shin, H-R 639

Shinkai, T 1142

Short, D 1145, 1709

Shu, X-O 1586

Sibold, S 307

Siboni, G 189

Siddik, ZH 1514

Siebels, M 463

Siemer, S 463

Sier, CFM 744

Siersema, PD 1180

Siewert, JR 1419

Silcocks, P 1576

Silva, RR 445

Simantov, R 581

Simi, L 879

Simiantonaki, N 210

Singh, D 1148

Sings, HL 1459

Sinha, AK 485

Siriwardena, BSMS 1396

Siu, LL 1148

Sjostrom, M 766

Skates, SJ 1124

Skliris, GP 616

Slabber, CF 1161

Slimani, N 406

Sluiter, W 1497

Smit, EF 470

Smit, WM 1334

Smith, AMH 6

Smith, IE 1632

Smith, J 841

Smith, PL 1689

Smith, T 1424

Snijders, PJF 96, 1593

Soder, M 463

Soejima, H 541

Soerjomataram, I 393

Sofronis, A 515

Soma, T 322

Sommer, HL 788

Sone, T 1390

Sonneveld, P 1497

Sørensen, HT 363, 934, 1609

Sørensen, M 416

Sørensen, NM 1114

Sörenson, S 966

Soufir, N 548

Soundararajan, CC 1250

Sousa, O 752

Spector, LG 1274

Spector, TD 520

Sperti, E 13

Spina, F 266

Spiro, A 431

Srolovitz, H 243

Staines, A 378

Stamps, AC 298

Stapley, S 1321

Stathopoulos, GP 587

Stebbing, J 1145, 1709

Steel, M 974

Steele, JC 1202

Steele, R 1490

Steers, G 298

Steinwall, M 1459

Stephens, R 966

Stern, PL 1258

Steyerberg, EW 1180

St-Jean, M 42

Stockton, DL 649

Stockwin, L 298

Stoehlmacher, J 1306

Sträter, R 991

Strohsnitter, W 107

Stroka, D 307

Stupp, R 710

Su, WC 159

Sugimoto, K 862

Sugita, Y 889

Sugiyama, S 561

Sukoh, N 998

Sullivan, F 974

Sun, CK 1050

Sun, LX 96

Sundstrøm, S 966

Surowiak, P 339

Sutani, A 1483

Sutcliffe, P 1195

Sutmuller, RPM 896

Swindell, R 1410

Symonds, P 735

Syrigos, K 587

Szeszenia-Dąbrowska, N 123

Tabata, M 1142

Tada, H 817

Taddeo, FJ 1459

Taguchi, H 1653

Tahmasebi, M 67

Taioli, E 1432

Tait, D 1632

Takahashi, H 1371

Takahashi, K 1244

Takahashi, N 1555

Takano, Y 1371

Takata, T 1396

Takayama, O 889

Takemasa, I 889

Takemoto, M 1142

Takeuchi, K 1642

Takeuchi, S 1653

Takeuchi, T 1653

Taki, T 817

Takigawa, N 1142

Takikawa, O 1555

Takizawa, H 75

Talbot, R 841

Tallini, G 1529

Tamaki, W 862

Tamms, GM 1459

Tampellini, M 13

Tamura, T 1390

Tan, S 1632

Tanaka, F 532

Tanaka, J 1562

Tanaka, T 1483, 1562

Tangoku, A 75

Tanimoto, M 1142

Tanzarella, S 181

Tappenden, P 1195

Tatematsu, M 1504

Taylor, BS 425

Te, V-C 390

Teixeira, MR 752

Telfer, C 921

ten Bokkel Huinink, WW 794

ten Hagen, TLM 1663

ten Hoor, KA 627

ten Kate, M 1497

Tenen, DG 554

Tenesa, A 239

Teng, NN 1314

Tepper, JE 260

Terauchi, M 1555

Terrett, JA 298

Teufel, A 210

Thar, TT 1379

Thatcher, N 822

Theillet, C 1439

Theys, J 1212

Thiele, CJ 879

Thijssen, B 729

Thomas, J 431

Thomas, R 1005

Thompson, D 233, 801

Thomson, R 1490

Thorburn, A 869

Thorpe, H 6

Tiffon, C 307

Tilakaratne, WM 1396

Tilanus, HW 1180

Tille, JC 1611

Tischkowitz, M 243

Tisdale, MJ 1028

Titus-Ernstoff, L 107

Tjønneland, A 406

Toita, T 862

Tomita, Y 1244

Tonge, D 172

Toonen, LWJ 896

Torr, EE 1202

Torrance, N 435

Torri, V 699

Tørring, N 1415

Tosoni, A 1155

Tourani, JM 1670

Trachsel, S 1474

Traina, A 1265

Traversari, C 181

Trayhurn, P 1028

Trétarre, B 944

Tretli, S 366

Trichopoulou, A 406

Tristram, A 226

Troisi, R 107, 1603

Trowman, R 457

Tsao, M-S 1148

Tschense, A 1280

Tse, LA 1269

Tsubono, Y 371

Tsuchiya, E 1070

Tsuji, I 371

Tsujino, T 889

Tsuneyama, K 1371

Tsunoda, S 322

Tucker, K 1448

Tumino, R 406, 1265

Tuncer, C 1545

Tung, ATY 1229

Turnbull, LW 801

Turnell, AS 555

Tyrer, J 525

Uccini, S 49

Uchida, Y 1483

Uchikado, Y 634

Uchino, H 601

Uchitomi, Y 146, 1610

Udagawa, K 1483

Ueno, M 607

Ueoka, H 1142

Underwood, MA 1234

Untch, M 788

Uozumi, J 541

Urban, R 1314

Urbanski, S 1020

Ursule, L 1439

Usadel, H 848

Ushijima, S 717

Utsunomiya, T 532

Vaccarella, S 96

Valle, JW 450

Vallès, X 355

Van Cutsem, E 450

van Dam, P 1362

Van de Perre, P 355

van de Velde, CJH 744

van de Vijver, MJ 1334

van den Berg, M 744

van den Brandt, PA 374

Van den Eynden, GG 1362

Van der Auwera, I 1362, 1611

van der Gaast, A 1180

van der Hout, AH 757

van der Reijden, JJ 744

van der Sangen, MJC 393

van der Velde, NM 1125

van der Wal, JBC 1497

van der Wall, E 1334

van der Zee, AGJ 627

van Dijk, BAC 374

van Eijck, CHJ 1497

van Hoesel, QGCM 1334

van Krieken, JHJM 1678

Van Laere, SJ 1362

van Lohuizen, M 1202

Van Marck, EA 1362

van Tiel, ST 1663

van Tinteren, H 1334

van Vliet, EPM 1180

Varambally, S 425

Varthalitis, J 587

Vassal, G 571

Vaughan, T 1212

vd Tol, A 470

Veiga, I 752

Vekhoff, A 253

Velasco, G 197

Velten, M 944

Veltkamp, SA 729

Verdecchia, L 445

Vermeulen, J 1326

Vermeulen, PB 1362, 1611

Verneris, MR 1274

Verspaget, HW 744

Verweij, F 289

Vessey, M 385

Vettorazzi, M 1265

Vezyraki, P 674

Viacava, P 204

Vias, M 245

Villa, LL 1459

Villanova, G 788

Vilsvik, J 966

Vinjamuri, S 1424

Vinson, GP 67

Vivekanandhan, S 1250

Vlahovic, G 1013

Voelter, V 710

Voest, EE 1334

Vogl, UM 691

von Krogh, G 1459

von Plessen, C 966

Voorzanger-Rousselot, N 506

Vornanen, M 378

Vorobiof, D 1161

Voso, MT 1108

Vowler, SL 314

Vuilleumier, H 710

Vujaskovic, Z 1013

Vysny, H 974

Wada, H 607

Wada, T 561

Wager, M 1062

Wagner, S 991

Wald, NJ 112

Walker, C 1424

Walker, L 233

Walker, LG 801

Wallerand, H 1455

Wallin, K-L 331

Walter, A 1081

Wang, H 1696

Wang, JH 247

Wang, X 475

Wang, Y 829, 1087

Ward, DG 1379

Ward, R 914

Ward, RL 1239

Ward, SE 27

Warmuth-Metz, M 991

Warnke, PC 1424

Warren, R 801

Watanabe, G 322

Watanabe, T 1562

Watanabe, Y 1142

Watkins, J 683

Watson, AJM 35

Watson, MS 435

Watson, PH 616

Webb, EL 1047

Webster, L 829

Wehler, T 210

Wei, W 1379

Weih, L 667

Weikert, S 1701

Weimann, R 307

Weiss, HA 355

Weissfeld, JL 1432

Weller, RE 1537

Wells, A 164

Welman, A 1410

Wernli, M 1342

Weston, C 571

Wezenberg, SJ 1678

Wheeler, P 56

Whitaker, NJ 1708

White, JS 1432

White, SC 822

Whiteside, TL 764

Wibom, C 766

Wigmore, SJ 1568

Wild, SH 424

Willers, R 1081

Williams, M 1490

Williams, R 914, 1239

Williamson, SE 35

Wilson, B 435

Wilson, GD 928

Wilson, GR 1410

Wilson, MS 1258

Wise, LA 107

Witte, D 782

Wiuf, C 1415

Wojnar, A 339

Wojtukiewicz, M 788

Wolff, JEA 991

Wolk, A 1277

Wolkenstein, P 548

Wong, S-L 1269

Wong, Y-C 475

Woods, LM 1296

Wordsworth, S 435

Workman, G 1092

Wouters, BG 1212

Wright, D 62

Wu, MF 159

Wu, T 112

Wülfing, P 347

Wü rtz, SØ 1114

Wynford-Thomas, D 496

Xia, C 581

Xiang, Y-B 1586

Xiao, ZJ 282

Xu, W-H 1586

Xu, X 889

Xue, WC 1087

Yamada, K 1070

Yamamoto, E 1555

Yamamoto, H 717, 889

Yamamoto, N 677

Yamana, K 1244

Yamazaki, K 998

Yan, B 1696

Yang, Y 1653

Yang, YC 1455

Yao, WQ 1593

Yap, A 829

Yasumitsu, T 817

Yatabe, Y 1504

Yates, P 667

Ye, W 118

Yee, D 1220

Yeend, SJ 1309

Yeh, KH 159

Yokomise, H 607

Yokota, A 1354

Yokouchi, H 998

Yokoyama, H 1504

Yonei, T 1142

Yoo, K-Y 639

Yoo, NC 1637

Yoshida, N 1555

Yoshihara, M 1070

Yoshii, Y 862

Young, A 1145, 1709

Young, D 974

Young, LS 1202, 1404

Youssef, MMS 735

Yu, IT-S 1269

Yuasa, T 1354

Zabel, M 339

Zahlten-Hinguranage, A 782

Zalcberg, J 829

Zatonski, W 123

Zecca, E 1121

Zehetgruber, H 691

Zehnder-Fjällman, AHM 272

Zeifang, F 782

Zeisberger, SM 272

Zhang, X 1220

Zhang, ZF 1455

Zhao, G-M 1586

Zhao, W 869

Zheng, H-C 1371

Zheng, W 1586

Zhou, W 164

Zhu, T 282

Zielinski, CC 691

Zimmermann, J 506

Zingaretti, C 445

Zino, S 1056

Ziras, N 587

Zohar, S 253

Zola, P 266

Zollino, M 1108

Zorzi, M 1265

Zouhair, A 710

